# Nanobody-horseradish peroxidase fusion protein as an ultrasensitive probe to detect antibodies against Newcastle disease virus in the immunoassay

**DOI:** 10.1186/s12951-019-0468-0

**Published:** 2019-03-01

**Authors:** Yamin Sheng, Kun Wang, Qizhong Lu, Pinpin Ji, Baoyuan Liu, Jiahong Zhu, Qingyuan Liu, Yani Sun, Jingfei Zhang, En-Min Zhou, Qin Zhao

**Affiliations:** 1 Department of Preventive Veterinary Medicine, College of Veterinary Medicine, Northwest A&F University, Yangling, 712100 Shaanxi China; 2 Scientific Observing and Experimental Station of Veterinary Pharmacology and Diagnostic Technology, Ministry of Agriculture, Yangling, 712100 Shaanxi China; 3 Xi’an Center for Animal Disease Control and Prevention, Xi’an, 710061 Shaanxi China

**Keywords:** Nanobody, Nanobody-HRP, Competitive ELISA, NDV, Antibody

## Abstract

**Background:**

Sensitive and specific antibodies can be used as essential probes to develop competitive enzyme-linked immunosorbent assay (cELISA). However, traditional antibodies are difficult to produce, only available in limited quantities, and ineffective as enzymatic labels. Nanobodies, which are single-domain antibodies (sdAbs), offer an alternative, more promising tool to circumvent these limitations. In the present work, a cELISA using nanobody-horseradish peroxidase (HRP) fusion protein firstly designed as a probe was developed for detecting anti-Newcastle disease virus (NDV) antibodies in chicken sera.

**Results:**

In the study, a platform for the rapid and simple production of nanobody-HRP fusion protein was constructed. First, a total of 9 anti-NDV-NP protein nanobodies were screened from a immunised Bactrian camel. Then, the Nb5-HRP fusions were produced with the platform and used for the first time as sensitive reagents for developing cELISA to detect anti-NDV antibodies. The cut-off value of the cELISA was 18%, and the sensitivity and specificity were respectively 100% and 98.6%. The HI test and commercial ELISA kit (IDEXX) separately agreed 97.83% and 98.1% with cELISA when testing clinical chicken sera and both agreed 100% when testing egg yolks. However, for detecting anti-NDV antibodies in the sequential sera from the challenged chickens, cELISA demonstrated to be more sensitive than the HI test and commercial ELISA kit. Moreover, a close correlation (R^2^ = 0.914) was found between the percent competitive inhibition values of cELISA and HI titers.

**Conclusions:**

A platform was successfully designed to easily and rapidly produce the nanobody-HRP fusion protein, which was the first time to be used as reagents for establishing cELISA. Results suggest that the platform supports the development of a cELISA with high sensitivity, simplicity, and rapid detection of anti-NDV antibodies. Overall, we believe that the platform based on nanobody-HRP fusions can be widely used for future investigations and treatment other diseases and viruses.

**Electronic supplementary material:**

The online version of this article (10.1186/s12951-019-0468-0) contains supplementary material, which is available to authorized users.

## Background

The enzyme-linked immunosorbent assay (ELISA) is a simple and rapid technique for detecting and quantitating antibodies or antigens that are immobilised to a solid surface based on an enzyme-labelled antibody [1]. ELISA offers commercial value in laboratory research, diagnosis of disease biomarkers, and quality control in various industries [2]. It is well known that enzyme-conjugated antibodies are the essential reagents for developing sensitive, specific, and reproducible ELISA. While traditional antibodies, including polyclonal and monoclonal antibodies, are universally used to label enzymes for developing ELISA [3], they are costly and require lengthy production times. For example, purification of monospecific antibodies and use of enzymatic labels, such as horseradish peroxidase (HRP) should be performed [4].

Recently, single-domain antibodies (sdAbs) derived from the variable domains of *Camelidae* heavy chain-only antibodies (VHH) have been extensively researched for diagnostic and therapeutic purposes [5, 6]. SdAbs, also known as nanobodies, retain the high affinity of the antigen-recognition site and are comprised of one variable domain containing a ~ 130 amino acid long chain [5, 7]. Nanobodies can be easily cloned and selected from immune or naïve VHH libraries due to their single-domain nature and strict monomeric behavior [8]. In addition, they are easy to genetically manipulate and derivate by coupling to reporters at a relatively low cost from a stored sequence [9]. Based on these features, nanobodies are becoming a more promising tool for the diagnosis and therapy of various diseases in comparison to conventional antibodies.

**Scheme 1:**
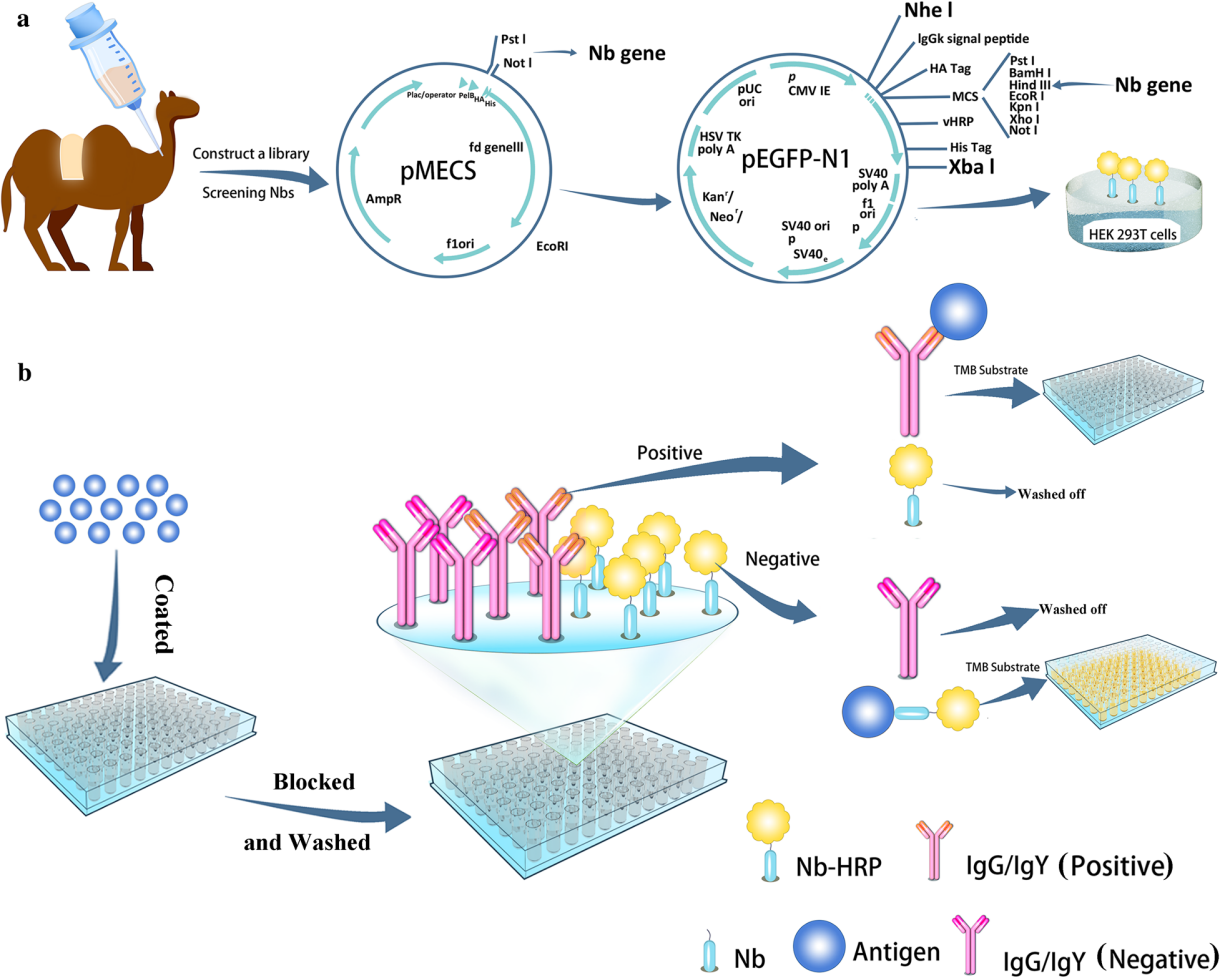
Schematic representation of screening the nanobodies from the immunised camel (**a**), the platform for expressing nanobody-HRP fusion proteins (**a**), and designation of the developed cELISA (**b**)

Newcastle disease has caused severe economic loss in the poultry industry worldwide due to the high costs of vaccinations and diagnostic laboratory investigations [10, 11]. The causative agent, Newcastle disease virus (NDV), is a single-strand, unsegmented negative-sense RNA virus comprised of six proteins: a large protein (L), hemagglutinin–neuraminidase (HN) protein, fusion protein (F), matrix protein (M), phosphoprotein (P), and nucleocapsid protein (NP) [12]. To date, the haemagglutination inhibition (HI) test is still the most widely used serological method for measuring anti-NDV antibody levels in poultry sera [13]. However, the assay often produces a high incidence of false positives in tested sera and requires a cumbersome operation [14]. In the present study, nanobodies against the NDV-NP protein were screened via phage display from a Bactrian camel immunised with the recombinant NDV-NP protein (Scheme 1a). Based on nanobodies against the NDV-NP protein, a platform was established to prepare nanobody-HRP fusion proteins, which were used for the first time as reagents to develop a sensitive, specific, and reproducible competitive ELISA (cELISA) for detecting anti-NDV antibodies (Scheme 1b). We believe that the simple, low-cost production of the nanobodies and nanobody-HRP fusions and their proposed application can be universally employed in the treatment of many other diseases and viruses.

## Materials and methods

### Cells, virus and vectors

HEK293T cells were cultured in Dulbecco’s Modified Eagle’s Medium (Life Technologies Corp, USA) and supplemented with 10% fetal bovine serum (FBS, Gibco, USA) at 37 °C in 5% CO_2_.

NDV strain LaSota was grown in the allantoic cavity of 8 to 9-day-old chicken embryonated eggs according to the previous procedures [15].

The pET-28a vector (Novagen, USA) was used for prokaryotic expression of the NDV-NP protein. The pMECS vector was used to construct the library of VHH [8]. The pEGFP-N1 vector (Clontech, Japan) was utilised as a backbone to construct the platform for nanobody-HRP fusions.

### Serum samples

To determine the cut-off value of cELISA in the study, a total of 244 sera from the specific pathogen free (SPF) chickens were used. For sensitivity and specificity, 355 positive and 368 negative chicken sera for anti-NDV antibodies were separately tested using cELISA. These positive and negative sera were confirmed via detection with a commercial ELISA kit (IDEXX, USA) according to the manual instructions and HI test based on the description in a previous study [16]. To validate the cELISA, a total of 220 serum samples were collected from 20 infected SPF chickens at 3, 4, 5, 6, 7, 10, 12, 14, 21, and 28 days post inoculation (dpi) with NDV strain LaSota. The HA titers of the challenged virus stock was 2^11^, and each SPF chicken was inoculated with 100 µL viral stock by the nasal route. In addition, these sequential sera were also detected using the commercial ELISA kit and HI test to compare results with those of cELISA. Meanwhile, 368 clinical chicken sera and 108 egg yolks from different flocks were also tested with the three methods for comparison. To determine if any cross reaction occurred with other antisera of the chicken virus tested using the developed cELISA, 103 clinical positive sera raised were investigated against other avian viruses, including the avian influenza virus (AIV) (n = 25), infectious bronchitis virus (IBV) (n = 35), infectious bursal disease virus (IBDV) (n = 25), and avian hepatitis E virus (avian HEV) (n = 18).

### Expression and purification of NDV-NP recombinant protein

The soluble NDV-NP recombinant proteins were expressed and purified based on the description provided by Kho et al. with some modifications [17]. Briefly, total RNA was extracted from 250 µL allantoic fluids containing the NDV stain LaSota using the Trizol reagent (Invitrogen, USA). The complete NP gene was amplified using TransScript II One-Step RT-PCR SuperMix (TransGen Biotech, China) according to the manual instructions with the primer pairs NDV-N-F and NDV-N-R (Additional file 1: Table S1). The PCR products were recovered from 1% agarose gel and ligated into the pET-28a vector with His tag (Novagen, USA). Then, the positive plasmid was transformed into *Escherichia coli* (*E. coli*) strain BL21 (DE3) cells. The NP protein expression was induced for 6 h by the addition of 1 mM isopropyl-β-d-thiogalactopyranoside (IPTG) when the *E. coli* cultures reached *A*_600_ around 0.6 to 0.8. The bacterial cells were centrifuged at 10,000*g* for 15 min at 4 °C. After sonication and centrifugation, the supernatant was loaded onto 1 mL His Trap excel (GE Healthcare Life Science, USA) connected to a ӒKTA purifier (GE Healthcare, USA). The protein was eluted with imidazole (500 mM) under a flow rate of 0.4 mL min^−1^. Finally, the expression, purification, and antigenicity of the recombinant NDV-NP protein were analysed by SDS-PAGE and Western blotting.

### Bactrian camel immunization and library construction

A 4-year-old male Bactrian camel was immunized by subcutaneous route with the recombinant NDV-NP proteins as described in previous studies [8, 18]. A mixture containing 1 mg purified recombinant NDV-NP protein (1 mg/mL) with the sample volume of Freund’s complete adjuvant was used for the first immunisation. For the next three immunisations, which were performed at 2-week intervals, only Freund’s incomplete adjuvant was used. The titration of the antibody against the NDV-NP protein in the serum samples from the last immunised Bactrian camel was detected with an indirect ELISA, using the recombinant NDV-NP proteins (200 ng/well) as the coating antigen. After incubation with camel serum, the second antibodies were detected by the mouse anti-camel antiserum, which was produced by immunizing the mice with purified camel IgG [18], and the HRP-conjugated goat anti-mouse IgG was used as the third antibody (Jackson ImmunoResearch Laboratories, USA). After the last immunisation, peripheral blood lymphocytes were isolated from a 250-mL blood sample by Leucosep^®^ tubes (Greiner Bio-One, Germany). Total RNA was extracted from the 10^7^ peripheral blood lymphocytes and used to synthesise the cDNA by reverse transcriptase. Subsequently, the VHH genes were amplified by nested PCR with the two primer pairs as described previously (Additional file 1: Table S1) [8]. The first PCR products, consisting of ~ 700 bp fragments with the first primer pairs, CALL001 and CALL002, were purified by agarose gel electrophoresis and used as the template for the second PCR. The final PCR products (approximately 400 bp) were amplified with the second primer pairs, VHH-FOR and VHH-REV, and then cloned into phagemid vector pMECS with the *Pst*I and *Not*I restriction sites. The recombinant phagemids were transformed into the freshly prepared electrocompetent *E. coli* TG1 cells. Cells were cultured on LB agar plates containing 2% glucose and 100 μg/mL ampicillin incubating at 37 °C overnight. On the 2nd day, the colonies were scraped from the plates, tested with primer pairs MP7 and GIII (Additional file 1: Table S1), and stored at − 80 °C in LB supplemented with 20% glycerol.

### Screening and identification of NDV-NP specific nanobodies

To select the NDV-NP nanobodies, three rounds of phage rescue and biopanning were performed as described previously [8, 18]. Briefly, phages were rescued via M13K07 phagemid purification. In biopanning, antigen-specific clones were enriched by binding them to the immobilised NDV-NP protein, followed by elution and re-propagation of phages. The plates (Nunc, Denmark) were coated with the purified 1 µg recombinant NDV-NP protein in the PBS buffer (pH 7.2) and incubated overnight at 4 °C. The plates were blocked with 200 μL of blocking buffer [PBS containing 10% (w/v) skim milk] at 37 °C for 2 h and washed with 0.5% PBS’T [PBS with 0.5% Tween-20 (v/v)]. Then, the rescuing recombinant phage particles were added to the plates and incubated at room temperature (RT) for 2 h. After being washed again with 0.5% PBS’T, the recombinant binding phages were simultaneously eluted by adding 100 μL Glycine–HCl buffer (pH 2.2) and neutralised with same volume of 1 M Tris buffer (pH 9.0). Next, a fresh growing culture of *E. coli* TG1 was infected with the eluted phages and amplified for further rounds of selection. After three or four rounds of biopanning, the enrichment of specific phage particles was calculated, and the 96 colonies were picked randomly for further analysis. Expressions of soluble VHHs with an HA-Tag in the 96 colonies were separately induced with IPTG (1 mM). All the recombinant VHHs-HA-Tag proteins were extracted from the periplasm and tested for their capacity to bind with the NDV-NP protein using an indirect ELISA with an anti-HA-Tag antibody (GenScript Biotech Corp, China). Finally, all VHH genes from the positive clones were sequenced, and the nanobodies were grouped according to their CDR3 sequence. In addition, to select the best nanobody for developing the following cELISA, blocking ELISA was used to determine if the positive chicken serum samples for anti-NDV antibodies blocked the different nanobodies bound with the NDV-NP protein. The procedure for block ELISA was similar to that of the indirect ELISA. However, the positive chicken serum samples were first added to the wells for 1 h incubation before anti-HA-Tag antibody was added.

### Production of nanobody-HRP fusion protein against NDV-NP protein

The platform of producing nanobody-HRP fusion protein was designed and constructed with some modifications based on a previous description [19]. DNA sequences, including a secreting signal sequence from the human Ig kappa chain, an HA tag, multiple cloning site (MCS), codon-optimized HRP, and His tag following a stop coding sequence, were synthesised and cloned into the multiple cloning sites of the commercial vector pCMV-N1-EGFP. The novelty vector was named pCMV-N1-HRP. Then, the selected positive VHH genes were obtained from the positive phagemid pMECS by digesting with both *Pst*I and *Not*I enzymes and ligated into the vector pCMV-N1-HRP also digested with the two enzymes. Then, the gene sequences of the nanobody were inserted between the HA tag and HRP sequences. The positive recombinant plasmids were confirmed by sequencing. To produce nanobody-HRP fusion protein, the mammalian cell line HEK293T was transfected with the constructing plasmid pEGFP-N1-HRP using polyetherimide (PEI, Polysciences Inc. Warrington, USA) agents. After the cells were transfected for 3 days, the medium containing secreted nanobody-HRP fusion proteins was harvested and filtered through 0.45-μm-pore cellulose acetate membranes for direct use.

The expression, amount, and titres of nanobody-HRP fusion protein in the medium were identified by indirect immunofluorescence (IFA) and ELISA with the medium or NDV-NP protein as the coated antigen. For IFA, after transfection 36 h, the cells were detected with anti-HA or His antibodies and were incubated using FITC-goat anti-mouse IgG antibodies (Jackson ImmunoResearch Laboratories, USA). Then, the cells were observed under fluorescence microscopy (Leica AF6000, Germany). For ELISA, the filtered medium was directly used for detection. For the amount of nanobody-HRP fusion protein in the medium, 100 and 200 µL of cultured medium after transfection were directly used to coat 96-well plates at 4 °C for overnight. Then, the plates were washed with PBS’T three times and added into tetramethylbenzidine (TMB) for a colorimetric reaction at RT for 15 min. Finally, the colorimetric reaction was stopped by adding 3 M H_2_SO_4_ (50 µL/well), and the OD_450nm_ values were read using an automated ELISA plate reader (Bio-Rad, USA). Meanwhile, another indirect ELISA was used to determine the titres of nanobody-HRP fusions against the NDV-NP protein in the medium. Briefly, the 96-well plates were coated with the purified recombinant NDV-NP protein (100 ng/well) using PBS buffer at 4 °C for overnight. Then, after washed three times, the plates were added into 100 µL of different dilutions of cultured medium and incubated for 1 h at RT. After another three times washings, TMB was added to the plates for a colorimetric reaction, then the OD_450nm_ values were read after the reaction was stopped.

### Development of the competitive ELISA using nanobody-HRP fusion protein as reagent

Based on the nanobody-HRP fusion protein, the cELISA was firstly designed. First, the optimal amount of coating antigen and dilution of selected nanobody-HRP fusions for the cELISA were determined using a checkerboard titration with direct ELISA. Different amounts of the coating antigen included 10, 20, 40, 80, 160, and 320 ng/well, and the dilution ratios of nanobody-HRP fusions were 1:1, 1:10, 1:100, 1:1000, and 1:10,000. The final conditions were determined from the reaction that produced approximately 1.0 of OD_450nm_ values in the direct ELISA.

The dilution of the tested chicken sera was also optimized. Four separate positive and negative chicken sera for anti-NDV IgG antibodies were diluted with 1:10, 1:20, 1:40, 1:80, 1:160, 1:320, 1:640, and 1:1280 for cELISA testing. The optimal amount of coated antigen and dilution of nanobody-HRP fusions were used. Then, the optimal serum dilution was determined when the smallest ratio of OD_450nm_ values between the positive and negative serum (P/N) were obtained.

The competition times of between testing sera antibody and nanobody-HRP fusions with antigen and colorimetric reaction were also optimised. The incubation times of the mixtures containing the nanobody-HRP fusions and the positive or negative sera with coated antigen were tested for 5, 10, 15, 20, 25, 30, 45, and 60 min. After TMB was added into the plates, 10 and 15 min were selected as the colorimetric reaction times. Using a checkerboard titration, the two optimal times were determined as those when the smallest ratio of P/N was obtained.

After optimising the above conditions, the 96-well ELISA plates were coated with the optimal amount of NDV-NP recombinant protein in the PBS (pH 7.2) buffer and incubated overnight at 4 °C or for 1 h at RT. Then, the plates were blocked with blocking buffer at RT for 1 h. After washed with PBS’T, the wells were added into 100 μL of testing mixtures containing the optimal dilutions of serum sample and nanobody-HRP fusions in the blocking buffer then incubated for optimal times at RT. After washed three times again, 100 μL TMB was added into each well, which were then incubated for optimal times at RT. As a final step, 3 M H_2_SO_4_ (50 μL/well) was used to stop the colorimetric reaction, and the OD_450nm_ values were read by an automated ELISA plate reader.

### Validation of the competitive ELISA

The following formula was used to calculate the percent competitive inhibition (PI): PI (%) = [1 − (OD_450nm_ value of testing serum sample/OD_450nm_ value of negative sample)] × 100%. The 244 negative sera samples from SPF chicken were used to determine the cut-off value and define positive and negative sera samples. All sera were detected with the cELISA, and the cut-off value was set at the mean PI of 244 negative serum samples plus 3 standard deviations (SD) to ensure 99% confidence for the negative sera samples within this range.

The sensitivity of cELISA was evaluated by testing 355 NDV-positive chicken sera from the flocks confirmed positive by the HI test and commercial ELISA kit. 220 sera from across the overall dpi range of the 20 challenged SPF chickens were also collected to determine the sensitivity of cELISA. In addition, two time dilutions (from 1:10 to 1:10,240) of 30 positive chicken sera for anti-NDV antibodies were also detected with the cELISA to determine the lowest detection limit.

To determine the specificity of cELISA, 368 clinical chicken sera from non-infected and immunised flocks were tested. The flock was confirmed to be non-infected by virus isolation in chicken embryonated eggs from fecal samples and by antibody detection with the HI test from serum samples. Meanwhile, the cross-competing assay between the nanobody-HRP fusions and other antibodies against the chicken virus, including AIV, IBDV, IBV, and avian HEV, were also evaluated.

The reproducibility of cELISA was evaluated by testing three positive and three negative samples selected from the clinical chicken sera. These six samples were used to perform the intra-assay and inter-assay variabilities. The coefficient of variation (CV) was used to evaluate the inter-assay variation (between plates) and the intra-assay variation (within a plate). Each sample was tested using three different plates tested on different occasions to determine the inter-assay CV, and three replicates within each plate were used to calculate the intra-assay CV.

### Comparisons of competitive ELISA with HI test and with commercial ELISA kit

The 368 clinical chicken sera and 220 sequential sera from the different dpi of challenged SPF chickens were tested using the developed cELISA, HI test, and commercial ELISA kit. Then, the coincidence rates between cELISA, HI test, and the commercial ELISA kit were calculated using Microsoft Excel’s CORREL function. In addition, the 82 positive chicken sera were both detected with cELISA and HI test. Based on the PI values of cELISA and HI titres of these sera, the correlation between cELISA and HI test was calculated with the Microsoft Excel program described in a previous study [20]. Further, to obtain the correlation between the cELISA and commercial ELISA kit, two time dilutions (1:10 to 1:10,240) of 98 positive chicken sera were also detected both with cELISA and the commercial ELISA kit. Based on the PI values of cELISA and S/P values of the commercial ELISA kit, a correlation was also calculated with the Microsoft Excel program.

### Statistical analysis

Student’s t-test and Kappa index values were calculated to estimate the differences in antigen binding blocking exhibited by the different nanobody-HRP fusions, as well as the coincidence between cELISA and the HI test and between cELISA and the commercial ELISA kit [21]. These calculations were performed using SPSS software (Version 20, http://www.spss.com.cn).

## Results

### Production of the soluble NDV-NP recombinant protein

**Fig. 1:**
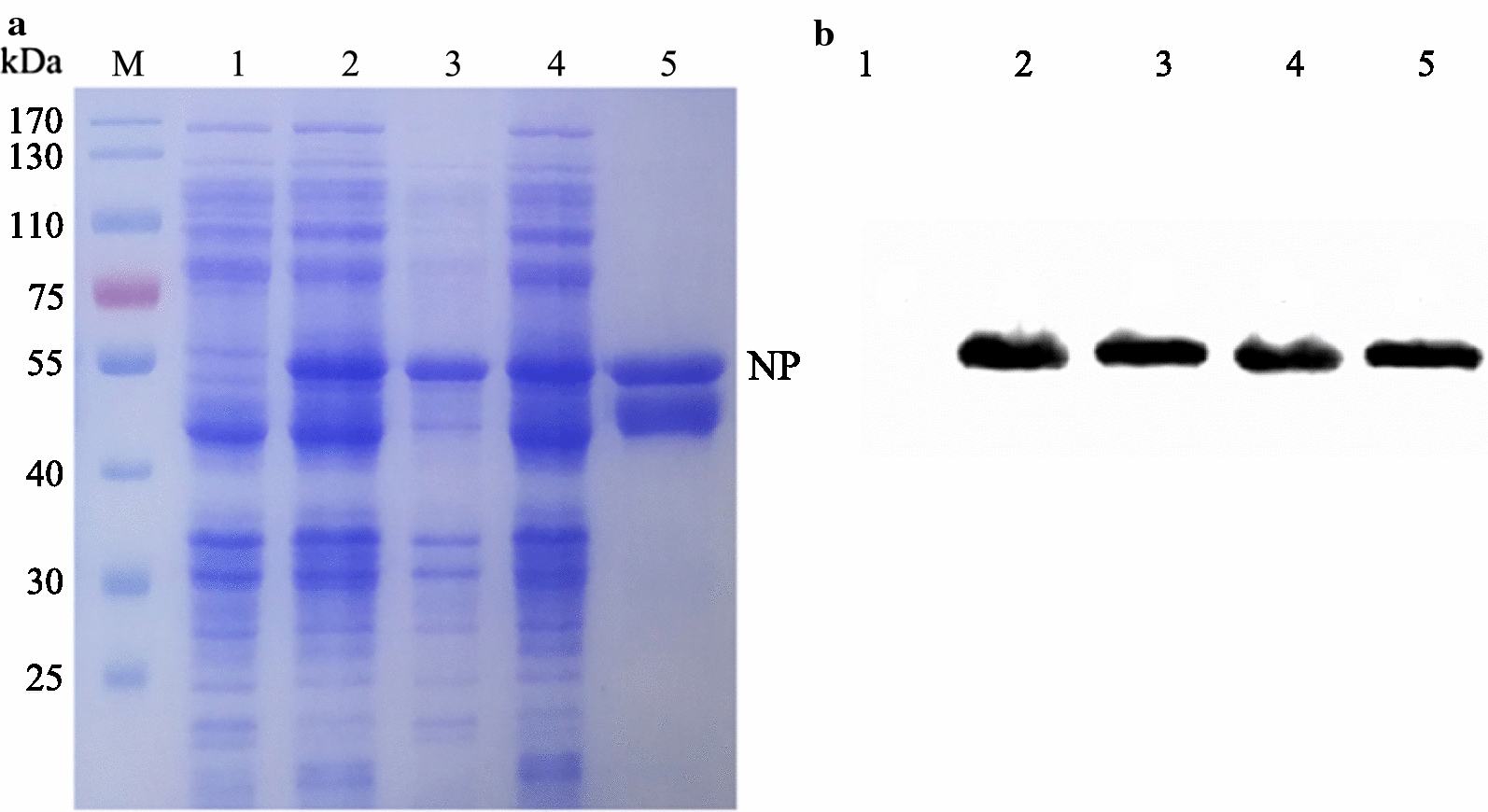
Expression, purification, and identification of the recombinant NDV-NP protein from the LaSota strain. **a** SDS-PAGE analysis, M, protein molecular markers; lane 1, pET-28a vector control; lane 2, bacterial lysates of the recombinant protein; lane 3, inclution body; lane 4, soluble protien; lane 5, purified protein. **b** Antigenic analysis of Western blotting, lane 1–5: same as **a**, reaction with the positive chicken sera for anti-NDV antibodies

After the recombinant proteins were induced for expression and purification, SDS-PAGE analysis showed that the recombinant NDV-NP proteins were successfully expressed in soluble form with the expected size 55 kDa, and the highest purity of recombinant protein was obtained (Fig. 1a). In addition, Western blotting analysis revealed that the recombinant NDV-NP protein reacted with the positive chicken sera for anti-NDV antibodies (Fig. 1b). The purified recombinant NDV-NP proteins were used as immunogens to immunise the camel and to coat antigens for screening the nanobodies and develop cELISA.

### Construction of a phage display VHH library

**Fig. 2:**
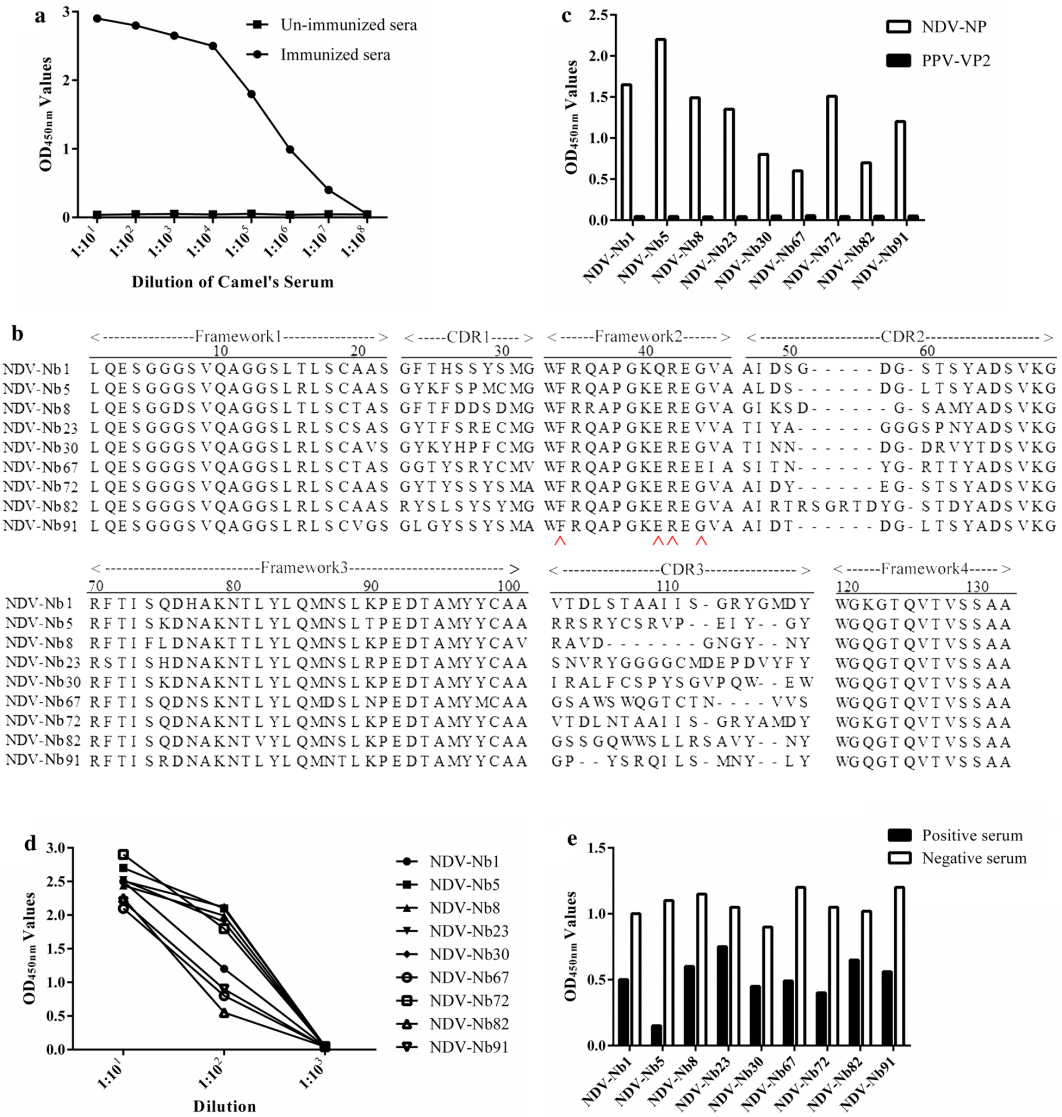
Screening the nanobodies against the NDV-NP protein. **a** Titres of antibodies against NDV-NP protein in the sera from the camel after the fifth immunisation. **b** Alignment of amino acid sequence of 9 screened nanobodies. Numbering and CDRs are according to the previous methods [4]. The residues at positions 37, 44, 45, and 47 are indicated by red arrows. **c** Specific reactions between the 9 screened nanobodies and NDV-NP protein. **d** Titration of the 9 screened nanobodies binding with the NDV-NP protein. **e** Analysis of the 9 screened nanobodies blocking the binding between the chicken sera and NDV-NP protein by blocking ELISA

After the last immunisation, the titres of antibodies against the NDV-NP protein in the camel serum were determined by indirect ELISA and reached 1:10^7^ (Fig. 2a), indicating that the camel produced a good immune response to the NDV-NP protein. According to previously described methods, a phage display VHH library against NDV-NP protein, consisting of approximately 3 × 10^9^ individual colonies, was successfully constructed from B lymphocyte cDNA encoding VHHs. Over 97.8% of these colonies had an insert corresponding to the size of a VHH gene (approximately 700 bp), as determined by colony PCR (Additional file 1: Fig. S1). Subsequently, a total of 96 clones were randomly selected and sequenced. Each clone was shown to contain a distinct VHH sequence, which confirmed the heterogeneity of the individual clones from the library (data not shown).

### Screening and sequencing of nanobodies against NDV-NP protein

After the third round of panning, a strong enrichment of phage particles carrying specific VHHs against NDV-NP protein was observed (Table 1). Then, the periplasmic extracts from 96 individual colonies were expressed and screened for binding to NDV-NP protein in an indirect ELISA. Out of which, 95 fragments were identified for specific binding to the NDV-NP protein (Additional file 1: Fig. S2) and were sequenced. Subsequently, the 95 sequences analysis revealed that a total of 9 nanobodies were screened and identified based on the CDR3 region (Fig. 2b). For amino acid sequences analysis, conserved residues at 37, 44, 45, and 47 positions (located on VH-VL interface region of VHs) from the 9 nanobodies were determined to be hydrophilic amino acids (Fig. 2b). In addition, the indirect ELISA results for specific binding showed that all 9 nanobodies could react with NDV-NP, but not with the control protein PPV-VP2 (Fig. 2c). The PPV-VP2 was expressed and purified using the same vector pET-28a and method as those for NDV-NP, which also has a 6 × His-Tag, eliminating the possibility that the nanobodies may recognise the 6 × His region. In addition, of the 9 nanobodies, NDV-Nb1, -Nb5, -Nb8, -Nb23, -Nb72, and -Nb91 showed the highest binding activity (Fig. 2d). Finally, to select the best nanobody from the 9 to develop cELISA, blocking ELISA was firstly used to analyse the positive chicken sera blocking the 9 nanobodies binding with the NDV-NP protein. The results suggested that the blocking rate for nanobody NDV-Nb5 was the highest (Fig. 2e); therefore, NDV-Nb5 was selected, designed, and expressed for further development of cELISA using nanobody-HRP fusions as reagents.

**Table 1:** Enrichment of nanobodies against NDV-NP protein from the phages during three rounds of panning

Round of panning	Phage input (PFU/well)	Phage output (PFU/well)	Recovery rate	Enrichment
1st round	5 × 10^10^	7 × 10^3^	1.4 × 10^−7^	7
2nd round	5 × 10^10^	1.17 × 10^7^	2.34 × 10^−4^	4.5 × 10^1^
3rd round	5 × 10^10^	1.14 × 10^7^	5 × 10^−4^	2.19 × 10^3^

### Design and expression of NDV-Nb5-HRP fusion proteins in HEK293T cells

**Fig. 3:**
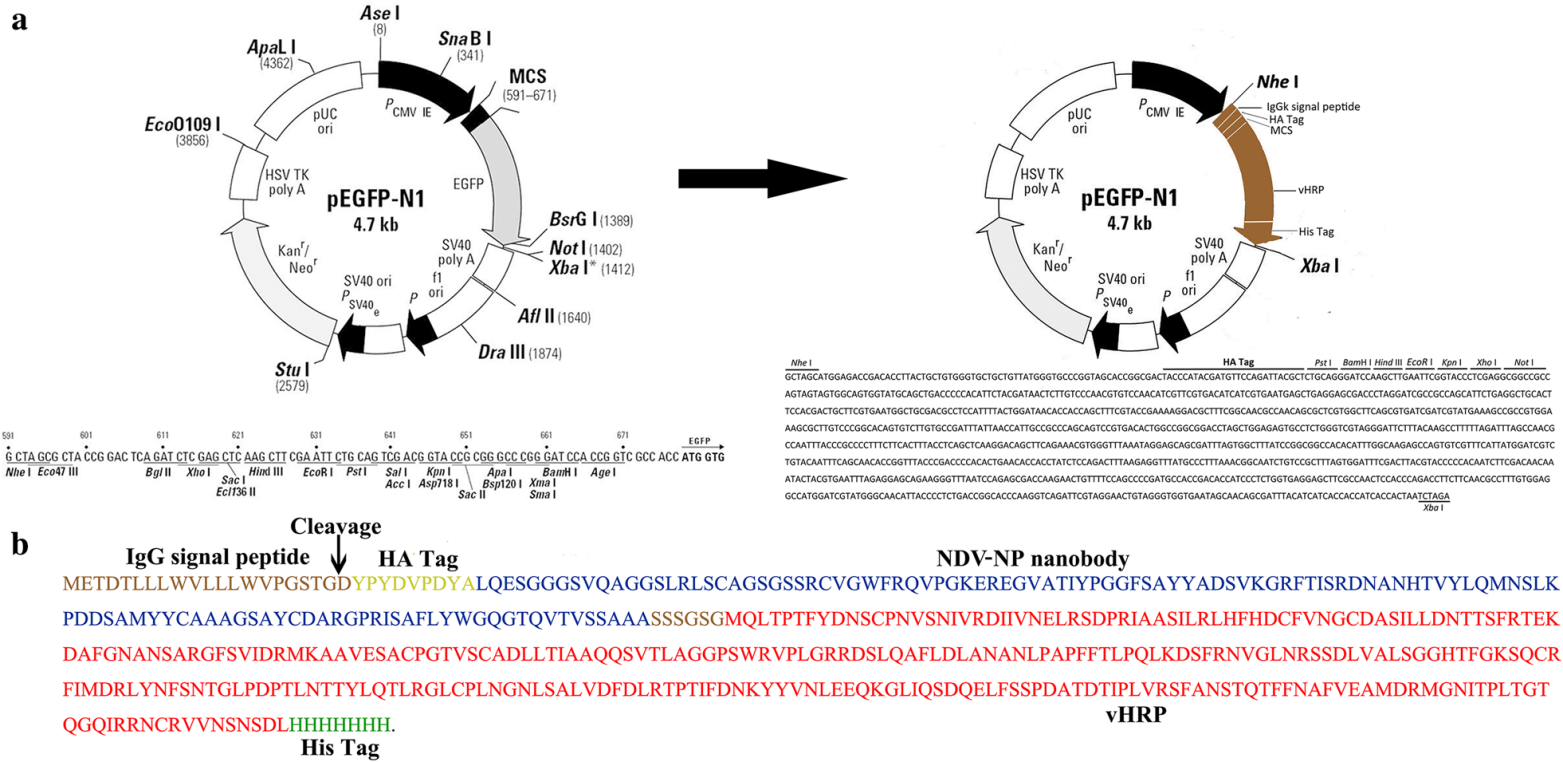
Construction of the platform for expressing the nanobody-HRP fusion protein. **a** Schematic presentation of the commercial vector pCMV-N1-EGFP changed into the vector to insert the main genes encoding vHRP. **b** Amino acid sequences encoded by the instering gene into the pCMV-N1-EGFP vector. Amino acids in the brown, yellow, blue, red, and green show the IgG signal peptide, HA tag, NDV-Nb5, HRP, and His tag, respectively

The DNA sequences, including a signal sequence from the human Ig kappa chain, HA tag, MCS, codon-optimized HRP, His tag, and stop codon, were inserted between the *Nhe*I and *Xba*I enzyme sites in the commercial pCMV-N1-EGFP vector (Fig. 3a). Then, the genes encoding NDV-Nb5 obtained from the pMECS vector using the *Pst*I and *Not*I enzyme digestion were inserted into the MCS region of the modified vector with same enzyme digestions (Fig. 3a). Subsequently, the deducing amino acids of all genes in the modified vector were expressed in the HEK293T cells, as shown in Fig. 3b.

**Fig. 4:**
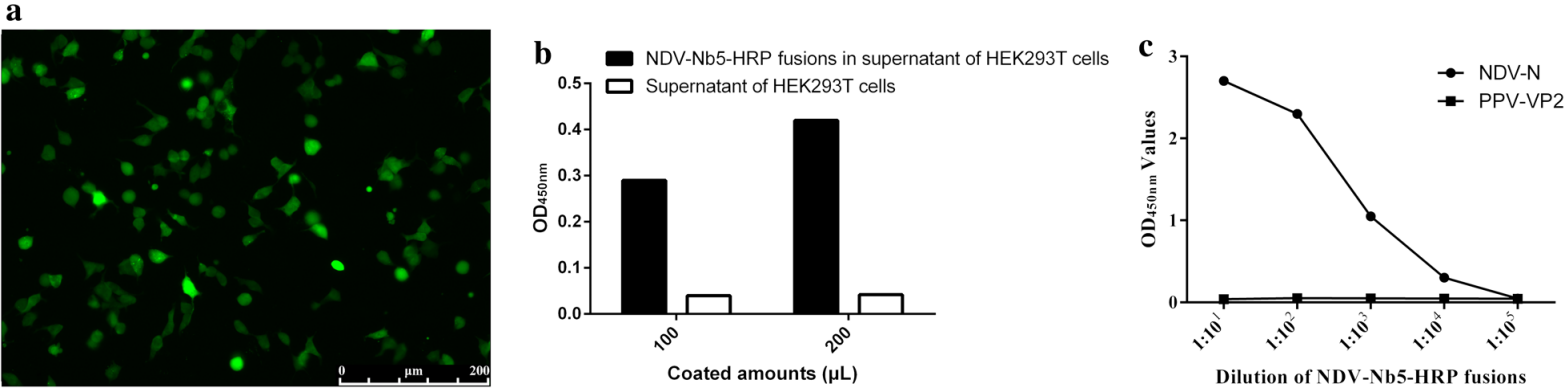
Identification of NDV-Nb5-HRP fusion protein expression and secretion in the HEK293T cells. **a** Detection of NDV-Nb5-HRP expressed in the HEK293T cells with the anti-HA mAb as the first antibody by IFA. **b** Detection of the HRP activity in the NDV-Nb5-HRP fusions secreted into the culture medium of HEK293T cells. **c** Detection of the NDV-Nb5-HRP reaction with the NDV-NP protein using indirect ELISA

After the recombinant plasmid was transfected into the HEK293T cells, the IFA results revealed that the NDV-Nb5-HRP fusions were successfully expressed in the cells with anti-HA mAb for detection (Fig. 4a). The results of direct ELISA using the medium containing NDV-Nb5-HRP fusions as the coating antigen showed that the fusions can be coloured by directly adding TMB, suggesting that HRP can expressed in the cells and retain bioactivity (Fig. 4b). In addition, the results of indirect ELISA using the NDV-NP protein as the coating antigen suggested that the NDV-Nb5-HRP fusions in the medium can still bind with the target protein (Fig. 4c). Meanwhile, the amount of NDV-Nb5-HRP fusions in the medium can be defined that the titres were 1:1000 (OD_450nm_ value was approximately 1.0) when the OD_450nm_ value was approximately 0.4 using the medium as the directly coated antigen.

### Competitive ELISA using the NDV-Nb5-HRP fusion proteins as reagents

The results of a checkerboard titration assay showed that the optimal amount of NDV-NP protein was 80 ng/well, and the dilution of NDV-Nb5-HRP fusions was 1:10^3^ (Table 2). The different dilutions of testing chicken sera in the cELISA with positive and negative sera revealed that the best dilution of chicken sera was 1:20 for the cELISA (Table 3). The results of a checkerboard assay for determining the optimal times for incubation of mixtures containing chicken sera and NDV-Nb5-HRP fusions with antigens and after adding TMB showed that the P/N value was smallest when the incubating time of the mixture with NDV-NP protein was 30 min, and the colorimetric reaction time was 10 min (Table 4).

**Table 2:** Optimised amount of NDV-NP protein as the coating antigen and dilution of NDV-Nb5-HRP fusions in medium using the indirect ELISA

Different amounts of NDV-NP proteins (ng)	Different dilutions of NDV-Nb5-HRP fusions in the medium			
	10^−1^	10^−2^	10^−3^	10^−4^
10	0.3315	0.237	0.11	0.058
20	0.798	0.5115	0.185	0.0595
40	1.822	0.847	0.3705	0.0515
80	2.5565	2.188	1.0945	0.185
160	2.7785	2.4215	1.317	0.223
320	2.908	2.643	1.5805	0.3005
640	3.05	2.8035	1.846	0.3505

The optimal amount of NDV-NP protein and dilution of NDV-Nb5-HRP were selected when the OD_450nm_ value of the indirect ELISA was approximately 1.0

**Table 3:** Optimised dilution of tested chicken sera for cELISA

No. serum	Sera type	1:10	1:20	1:40	1:80	1:160	1:320	1:640	1:1280
1	Positive	0.14	0.16	0.24	0.44	0.73	0.97	0.98	0.93
	Negative	0.90	1.03	1.06	1.06	1.02	1.09	1.02	1.08
	P/N	0.15	0.16	0.23	0.42	0.72	0.89	0.96	0.87
2	Positive	0.10	0.13	0.24	0.50	0.80	1.00	1.12	1.03
	Negative	0.90	1.03	1.00	1.02	0.99	1.01	1.13	1.16
	P/N	0.11	0.13	0.24	0.48	0.81	0.99	0.99	0.89
3	Positive	0.08	0.11	0.16	0.38	0.73	0.94	1.11	1.12
	Negative	1.01	1.09	1.10	1.11	1.09	1.17	1.14	1.20
	P/N	0.08	0.10	0.15	0.35	0.67	0.80	0.97	0.94
4	Positive	0.08	0.10	0.14	0.24	0.55	0.84	0.96	1.02
	Negative	0.89	1.00	0.90	1.00	1.02	0.89	1.08	1.06
	P/N	0.09	0.10	0.16	0.24	0.54	0.95	0.89	0.96

Four positive and negative chicken sera were separately used for cELISA detection. The dilutions of sera were 1:10, 1:20, 1:40, 1:80, 1:160, 1:320, 1:640, and 1:1280. The best dilution was selected when the OD_450nm_ value of positive to negative (P/N) sera was smallest

**Table 4:** Optimised incubation time of the mixture containing chicken sera and NDV-Nb5-HRP fusions incubated with the antigen and optimal time for the colorimetric reaction after adding TMB using a checkerboard assay with cELISA

Times (min) of color reaction	Sera type	Incubation times (min) of chicken sera, NDV-Nb5-HRP fusions and antigens							
		5	10	15	20	25	30	45	60
10	Positive	0.69	0.86	0.52	0.54	0.48	0.24	0.24	0.29
	Negative	0.75	0.89	0.94	0.97	1.02	0.99	1.01	1.11
	P/N	0.92	0.97	0.55	0.56	0.47	0.24	0.23	0.26
15	Positive	0.70	0.63	0.50	0.47	0.39	0.25	0.26	0.29
	Negative	0.80	0.75	0.87	1.00	0.97	1.02	1.15	1.12
	P/N	0.86	0.84	0.57	0.47	0.40	0.25	0.23	0.26

After the above conditions were determined, the procedures of cELISA were performed as follows. First, the 96-well ELISA plates were coated with 80 ng/well NDV-NP recombinant protein in the PBS buffer and incubated overnight at 4 °C or for 1 h at RT. Then, the plates were blocked with 200 µL blocking buffer at RT for 1 h. After washed with PBS’T, the 100 μL of testing mixtures containing 5 µL testing chicken serum sample and 95 µL NDV-Nb5-HRP fusions with 1:1000 dilution in the blocking buffer were added and incubated at RT for 30 min. After washed three times again, 100 μL TMB was added into each well, which were then incubated for 10 min at RT. As a final step, 3 M H_2_SO_4_ (50 μL/well) was used to stop the colorimetric reaction, and the OD_450nm_ value was read by an automated ELISA plate reader. Based on these procedures, the operation of cELISA is simple, the time is rapid and the cost is less compared with the previous assays.

### Cut-off values for the competitive ELISA

The 244 SPF chicken sera were used to determine the cut-off value for cELISA. The results showed that the average PI (X) value of the 244 negative chicken sera was 3.0% with an SD of 5.0%, and the cut-off value for cELISA was 18% (3.0% + 3SD). The cut-off value was considered negative, or positive, if the PI values of the tested chicken sera with the cELISA were less than, or more than, 18%, respectively.

### Sensitivity, specificity and reproducibility of the competitive ELISA

**Fig. 5:**
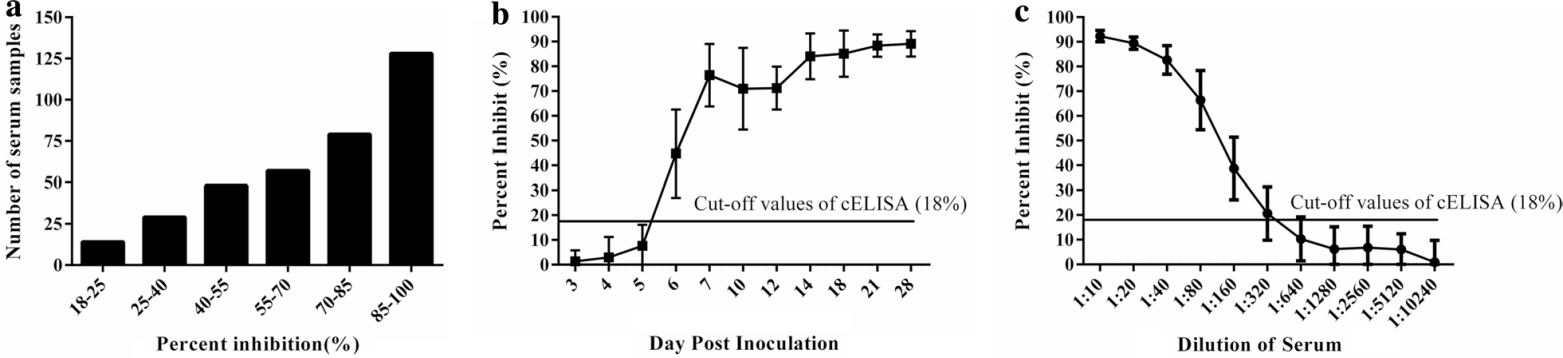
Sensitivity of the cELISA for detecting anti-NDV antibodies using the NDV-Nb5-HRP fusion protein as a probe. **a** Distribution of the PI values from the cELISA for detecting the clinical positive chicken sera for anti-NDV antibodies. **b** Detection of antibodies against NDV in the serial sera from the challenged SPF chickens with NDV strain LaSota with the cELISA. **c** Determination of the largest dilution of positive chicken sera for anti-NDV antibodies

For determining the sensitivity of the cELISA, the 355 clinical positive chicken sera were all positive via detection with cELISA with PI values ranging 20–95% (Fig. 5a). The PI values of 128 serum samples were greater than 85%, and only 14 samples had PI values from 18 to 25% (Fig. 5a). Thus, the sensitivity of cELISA for the tested clinical chicken sera was 100%. For the 220 sequential sera, the results revealed that all 20 SPF chickens seroconverted at 6 wpi using cELISA for detection, and until 28 dpi, all chickens were still positive for anti-NDV antibodies (Fig. 5b). The antibodies against NDV can be first detected from the 5 dpi sera. For the different dilutions of the 10 positive chicken sera, all sera at the dilution of 1:1280 were negative using cELISA, while only 3 samples were negative when the dilution was 1:320 (Fig. 5c). Therefore, for the most positive chicken serum samples, the largest dilution was 1:160 for detecting the anti-NDV antibodies.

**Fig. 6:**
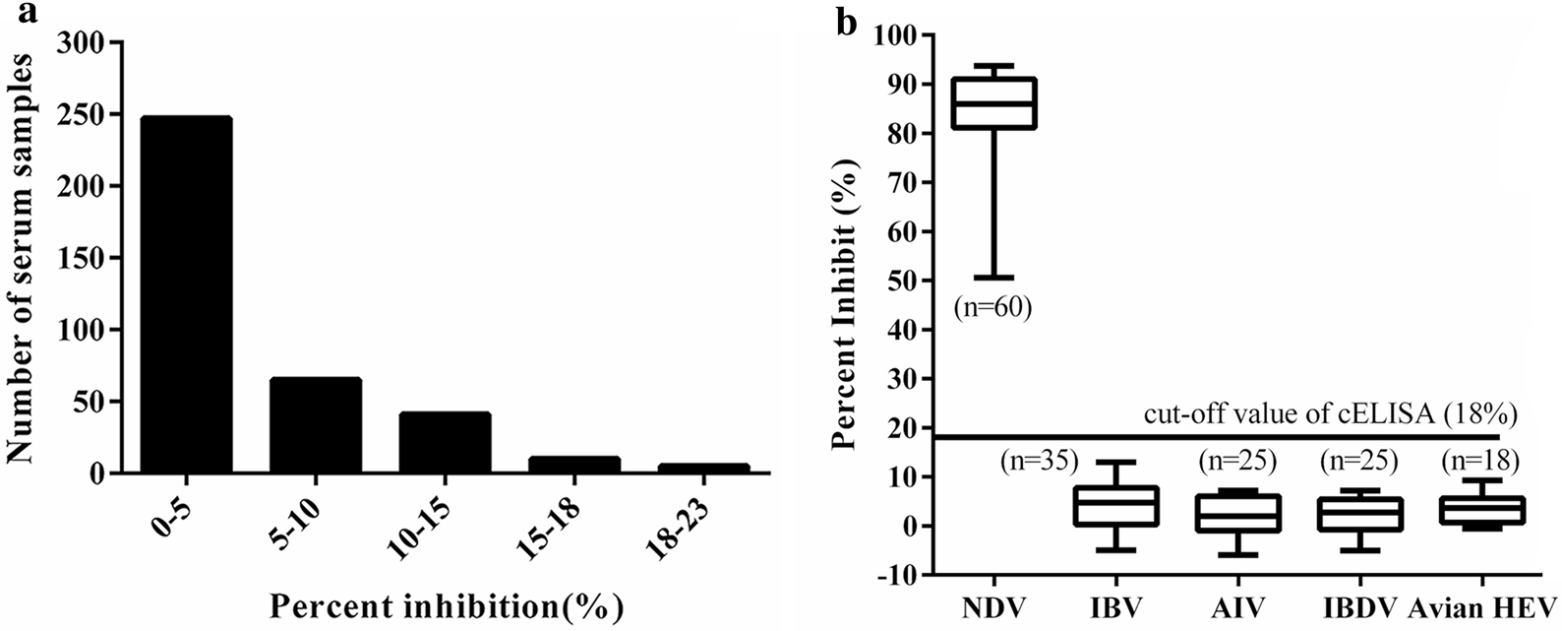
Specificity the cELISA for detecting anti-NDV antibodies using the NDV-Nb5-HRP fusions as a probe. **a** Distribution of the PI values from the cELISA to detect the clinical negative chicken sera for anti-NDV antibodies. **b** Evaluation of the cELISA detecting the antibodies against other chicken disease viruses, including IBV, AIV, IBDV, and avian HEV

For determining the specificity of cELISA, the results showed that 363 chicken sera were negative for anti-NDV antibodies detected with the cELISA with PI values ranging 2–18% (Fig. 6a). Thus, the specificity of cELISA was 98.6%. Furthermore, the antisera against other chicken viruses were negative with PI values from 3 to 10% (Fig. 6b).

By testing the 6 chicken sera in triplicate, the intra-assay CV of the PI were observed to range from 1.21 to 3.71% with a median value of 1.13%. When the 6 samples were tested in three plates at different times, the inter-assay CV of the PI ranged from 1.12 to 4.23% with a median value of 3.05%. These data indicate that the developed cELISA exhibits good reproducibility.

### Agreements of competitive ELISA with HI test and with commercial ELISA kit

After testing the 368 clinical chicken sera separately using the cELISA, HI test, and commercial ELISA kit, the positive rates of each method were 97.83% (360/368), 100% (368/368), and 99.18% (365/368) (Table 5), respectively. The results of cELISA and the HI test coincided in 360 of the 368 serum samples with an agreement rate of 97.83% (Table 5). Similarly, the results of cELISA and commercial ELISA kit agreed in 361 of the 368 ones with an agreement rate of 98.10% (Table 5). In addition, the 108 egg yolks were tested for anti-NDV antibodies separately with the cELISA, HI test, and commercial ELISA kit. The results showed that 96 egg yolks were positive for the anti-NDV antibodies with the three assays and with agreement rates of 100% (Table 5). To detect the clinical chicken sera and egg yolks, statistical analysis showed that cELISA had a high level of consistency with the HI test (Kappa = 0.742) and the commercial ELISA kit (Kappa = 0.793) (Table 5). No significant differences were found between cELISA and either the HI test or the commercial ELISA kit (all Kappa values were > 0.4).

**Table 5:** Comparisons of the developed cELISA with HI test and with commercial ELISA kit by detecting clinical chicken sera and egg yolks

Samples	Number	cELISA	Commercial ELISA kit		Agreement (%)	Kappa value	HI test		Agreement (%)	Kappa value
			+	^−^			+	−		
Chicken sera	360	+	359	1	97.83	0.793	360	0	98.10	0.742
	8	−	6	2			8	0		
Egg yolks	96	+	96	0	100		96	0	100	
	12	−	0	12			0	12		

Besides testing the clinical samples, the sequential sera from the challenged SPF chickens were also analysed to compare the sensitivity of the three assays. It was found that all collected sera at 6 dpi were positive for anti-NDV antibodies using cELISA, but only 4 samples were positive with the HI test and none were positive with the commercial ELISA kit (Table 6). At 5 dpi, 3 samples were positive with cELISA (Table 6), while at 10 dpi, all sera were positive using the cELISA and HI test, but only 4 sera were positive with the commercial ELISA kit (Table 6). At 12 dpi, these sera were positive using all three assays (Table 6), and until 28 dpi, these were still positive. The above results indicate that compared with the HI test and commercial ELISA kit, the sensitivity of the developed cELISA is higher with the optimised conditions.

**Table 6:** Comparisons of the cELISA with HI test and with commercial ELISA kit by detecting the sequential sera form the challenged SPF chickens with the NDV strain LaSota

Different assay	Different day post inoculation (dpi) (positive number/total number)										
	3	4	5	6	7	10	12	14	18	21	28
cELISA	0/20	0/20	3/20	20/20	20/20	20/20	20/20	20/20	20/20	20/20	20/20
HI test	0/20	0/20	0/20	4/20	15/20	20/20	20/20	20/20	20/20	20/20	20/20
Commercial ELISA kit	0/20	0/20	0/20	0/20	4/20	4/20	20/20	20/20	20/20	20/20	20/20

**Fig. 7:**
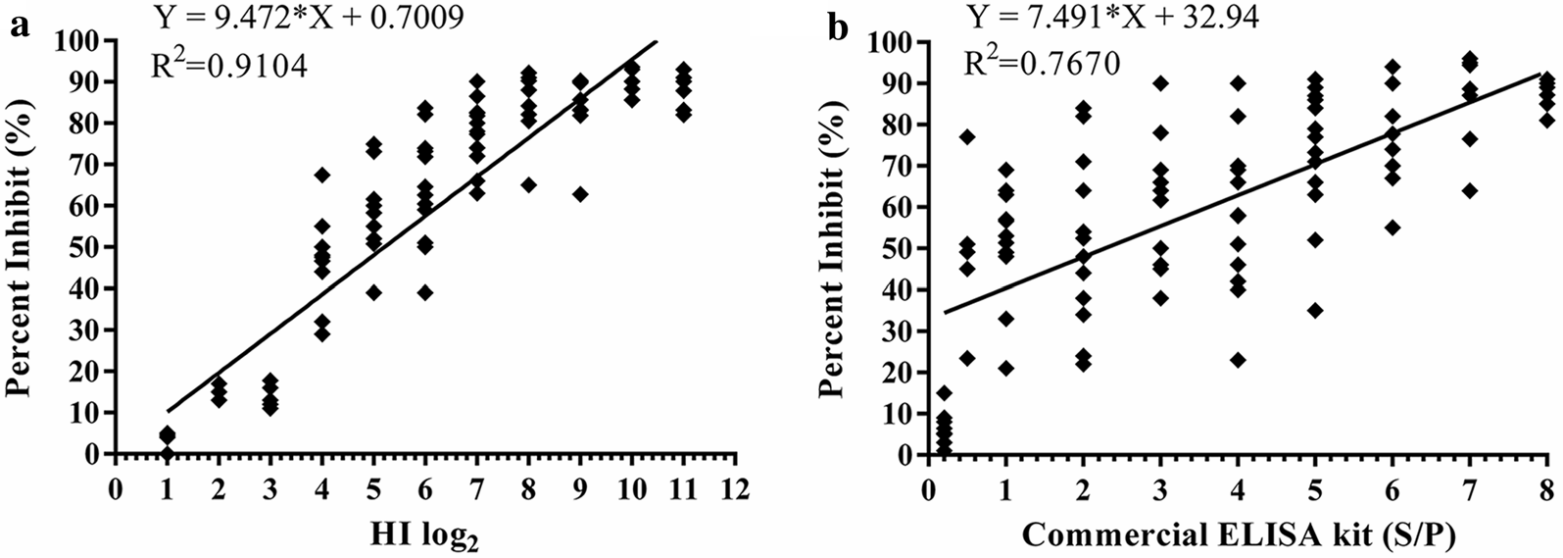
Correlation between cELISA and the HI test and the commercial ELISA kit (IDEXX). **a** Serum titers for antibodies against NDV detected between the cELISA (PI) and HI test (log2). **b** Titres of antibodies against NDV in different dilution serum samples detected between the cELISA (PI) and commercial ELISA kit test (S/P)

Clinically, the HI test has been widely used to detect anti-NDV antibodies from chicken sera for diagnosis purposes and evaluation of vaccine immunisation. A close correlation (R^2^ = 0.914) was found between the cELISA and HI titers (*P *< 0.0001) by a linear regression analysis (Fig. 7a). In addition, the correlation between S/P values of the commercial ELISA kit and PI values of cELISA were also performed using 98 clinical chicken sera. The results revealed a good correlation (R^2^ = 0.7670) with a *P* value of 0.0009.

## Discussion

For the different formats of ELISA, antibodies are the key. For example, cELISA utilises a mixture of an antibody-probe and free antibody in liquid phase to interact with plate-immobilised antigens [22]. Usually, the sensitivity and specificity of the assay are determined by the antibody-probe [22]. To date, traditional antibodies, including polyclonal and monoclonal antibodies, have been produced for developing ELISA [23, 24]. However, the high costs and difficult operations of conventional antibodies for production, purification, and enzyme labelling are already becoming a major burden for the further development of commercial ELISA kit [25, 26]. In the present study, a nanobody-HRP fusion platform was designed based on a previous study with some modifications [19], and fusion proteins were used for the first time as a probe to further develop cELISA (Scheme 1a). In the previous study, the platform was constructed by synthesizing sequences of a signal peptide, the different nanobodies and the HRP at each time [19]. In the present study, after the modified platform was constructed, the sequences of nanobodies can be easily digested from the phage vector pMECS and ligated into the modified platform. This modification cuts the costs. In addition, in the previous study the reporter-nanobody fusions were produced and used as reagents for developing immunohistochemical detection [19]. In the present study, the reporter-nanobody fusions were used for the first time to develop cELISA for detecting antibodies against NDV. The nanobodies-HRP can be easily produced by standard methods, which only include simple enzyme digestion, ligation, and transfection. If the cell lines stably expressing the nanobodies-HRP can be constructed, the production of nanobodies-HRP will be easier. Compared with the production of traditional antibodies for the commercial ELISA kit, nanobodies-HRP production is more simple and inexpensive. This is because the procedures for producing nanobodies-HRP omit purification and enzyme-labelling and only require standard methods in most laboratories. Moreover, no secondary antibody is required for detection. Most importantly, it was the first time to use the platform and methods for developing cELISA for detecting anti-NDV antibodies and can be rapidly used for other pathogens when nanobodies to additional antigens become available.

Newcastle disease causes a severe economic loss in the world’s poultry industry, leading to the development of many assays for diagnosis and surveillance of the disease [11]. Among these assays, the HI test is the most widely used serological method for measuring anti-NDV antibody levels and evaluating the effects of vaccine immunisation in poultry sera [13]. However, this test demands that chicken erythrocytes and NDV antigen must be first produced, increasing the difficultly of operating the assay [14]. For other ELISAs, the purification and enzyme-labelling of antibodies must be performed [27–29]. In addition, the cost times of HI test and other ELISAs are approximately 2 to 3 h. In the present study, based on an anti-NDV NP protein nanobody (Nb5), it was the first time to produce NDV-Nb5-HRP fusions using the proposed platform and then to develop a cELISA for detecting anti-NDV antibodies in chicken sera. Compared with the HI test and a commercial ELISA kit, the developed cELISA exhibits higher sensitivity, specificity, simplicity, and rapid detection times (only approximately 1 h). In addition, results revealed a close correlation between HI titres and PI values of the developed cELISA, which suggests that cELISA offers an alternative method to evaluate the effects of vaccine immunisation instead of the HI test. Therefore, it is proposed that the developed cELISA can be universally used to detect and measure anti-NDV antibody levels in chicken sera. For the future construction of cell lines stably expressing NDV-Nb5-HRP, our designed commercial cELISA kit can be easily produced and implemented in the poultry industry.

NDV can infect more than 200 species of birds [30], and mammals, like dogs, cats [31], and humans, are also susceptible to this virus [32]. However, sera from other species tend to induce a high incidence of false positive results with the HI test. To test anti-NDV antibodies in sera of different species using some commercial indirect ELISA kits, the second IgG antibodies of different species should be used. Theoretically, the developed cELISA in this study can be used to test anti-NDV antibodies in different species’ sera; yet, the testing of sera of more diverse species will be needed to further confirm this theoretical hypothesis.

## Conclusion

Nanobodies are commonly produced due to their advantages, especially against pathogens and antigens, and are used in current researches and as diagnostic tools. In the present study, nine NDV-NP specific nanobodies were produced from an immunised Bactrian camel. Based on these nanobodies, a platform was designed to easily and rapidly produce nanobody-HRP fusion proteins, which were implemented for the first time to develop a sensitive, specific, simple, and rapid cELISA for detecting anti-NDV antibodies in chicken sera. A close correlation was observed between the titres of the HI test and PI values of cELISA; thus, the platform can be easily used to develop the cELISA to detect anti-other pathogen antibodies when their specific nanobodies are available. Furthermore, the developed cELISA with NDV-Nb5 fusions offers a simpler, cost-effective and suitable method for the diagnosis and measurements of anti-NDV antibody levels in chicken sera compared to the widely used HI test.

## Additional file

**Additional file 1: Table S1.** Primer pairs in the study. **Fig. S1.** Evaluate the positive rate of the VHH library by colony PCR. **Fig. S2.** Analysis of the binding ability of recombinant nanobodies against NDV-NP protein by indirect ELISA.
