# Supplementary material for: Nanobody-horseradish peroxidase fusion protein as an ultrasensitive probe to detect antibodies against Newcastle disease virus in the immunoassay
Source: J Nanobiotechnology. 2019 Mar 1;17:35. doi: 10.1186/s12951-019-0468-0 (PMC6396497; doi:10.1186/s12951-019-0468-0)
Supplement: Supplementary file 1 — Additional file 1: Table S1. Primer pairs in the study. Fig. S1. Evaluate the positive rate of the VHH library by colony PCR. Fig. S2. Analysis of the binding ability of recombinant nanobodies against NDV-NP protein by indirect ELISA. [file 12951_2019_468_MOESM1_ESM.docx]

**Table S1 Primer pairs in the study.**

| **Names** | **Sequences (5’-3’)** | **Reference** |
| --- | --- | --- |
| NDV-NP-F | CGCATATGAGCAGCGTGTTCGATGAAT | [1] |
| NDV-NP-R | TACTCGAGGTAACCCCAGTCGGTATC |  |
| CALL001 | GTCCTGGCTGCTCTTCTACAAGG | [2] |
| CALL002 | GGTACGTGCTGTTGAACTGTTCC |  |
| VHH-FOR | GATGTGCAGCTGCAGGAGTCTGGRGGAGG | [2] |
| VHH-REV | CTAGTGCGGCCGCTGAGGAGACGGTGACCT GGGT |  |
| MP57 | TTATGCTTCCGGCTCGTATG | [2] |
| GIII | CCACAGACAGCCCTCATAG |  |

1. Kho CL, Tan WS, Yusoff K. Production of the nucleocapsid protein of Newcastle disease virus in Escherichia coli and its assembly into ring- and nucleocapsid-like particles. J Microbiol. 2001;39:293-9.

2. Vincke C, Gutierrez C, Wernery U, Devoogdt N, Hassanzadeh-Ghassabeh G, Muyldermans S. Generation of single domain antibody fragments derived from camelids and generation of manifold constructs. Methods Mol Biol. 2012;907:145-76.





**Fig. S1** Evaluate the positive rate of the VHH library by colony PCR.





**Fig. S2** Analysis of the binding ability of recombinant nanobodies against NDV-NP protein by indirect ELISA
